# A case–control study on autoimmune polyendocrine syndromes in patients with systemic lupus erythematosus

**DOI:** 10.1093/rheumatology/keaf320

**Published:** 2025-06-10

**Authors:** Elda Piovani, Giorgia Ingrid Gozzoli, Silvia Ebe Lucia Della Pina, Claudia Barison, Chiara Orlandi, Elisa Gatta, Cesare Tomasi, Micaela Fredi, Carlo Cappelli, Franco Franceschini

**Affiliations:** Rheumatology and Clinical Immunology Unit, ASST Spedali Civili of Brescia, Brescia, Italy; Department of Clinical and Experimental Sciences, University of Brescia, Brescia, Italy; Rheumatology and Clinical Immunology Unit, ASST Spedali Civili of Brescia, Brescia, Italy; Department of Clinical and Experimental Sciences, University of Brescia, Brescia, Italy; Rheumatology and Clinical Immunology Unit, ASST Spedali Civili of Brescia, Brescia, Italy; Department of Clinical and Experimental Sciences, University of Brescia, Brescia, Italy; Rheumatology and Clinical Immunology Unit, ASST Spedali Civili of Brescia, Brescia, Italy; Department of Clinical and Experimental Sciences, University of Brescia, Brescia, Italy; Rheumatology and Clinical Immunology Unit, ASST Spedali Civili of Brescia, Brescia, Italy; Department of Clinical and Experimental Sciences, University of Brescia, Brescia, Italy; Department of Clinical and Experimental Sciences, University of Brescia, Brescia, Italy; Endocrinology and Metabolism Unit, ASST Spedali Civili of Brescia, Brescia, Italy; Department of Clinical and Experimental Sciences, University of Brescia, Brescia, Italy; Rheumatology and Clinical Immunology Unit, ASST Spedali Civili of Brescia, Brescia, Italy; Department of Clinical and Experimental Sciences, University of Brescia, Brescia, Italy; Department of Clinical and Experimental Sciences, University of Brescia, Brescia, Italy; Endocrinology and Metabolism Unit, ASST Spedali Civili of Brescia, Brescia, Italy; Rheumatology and Clinical Immunology Unit, ASST Spedali Civili of Brescia, Brescia, Italy; Department of Clinical and Experimental Sciences, University of Brescia, Brescia, Italy

**Keywords:** systemic lupus erythematosus, endocrine, comorbidities, epidemiology, antiphospholipid syndrome

## Abstract

**Objectives:**

This study aimed to investigate the prevalence of autoimmune polyendocrine syndromes in patients with SLE and to assess whether autoimmune polyendocrine syndromes predict higher disease activity or worse outcomes.

**Methods:**

The clinical charts of 417 SLE patients (meeting SLICC 2012 and/or EULAR/ACR 2019 criteria)( referred to our centre between 2021 and 2023 were analysed. Autoimmune polyendocrine syndrome cases were identified using ORPHA definitions; 185 autoimmune polyendocrine syndrome–free SLE patients, randomly enrolled, served as controls. Demographic, clinical and serological data were collected.

**Results:**

Forty-seven of 417 (11%) SLE patients had another autoimmune disease affecting the glands that allowed the diagnosis of autoimmune polyendocrine syndrome: 39 were diagnosed with Hashimoto thyroiditis, 6 with Graves’ disease, and 3 with type 1 diabetes mellitus. Forty-five were affected by autoimmune polyendocrine syndrome type 3, and 2 by autoimmune polyendocrine syndrome type 4; no patients were diagnosed with autoimmune polyendocrine syndrome types 1 or 2. A comparison between the autoimmune polyendocrine syndrome–positive and –negative patients revealed no significant differences in clinical or serological features. At the last evaluation, ∼80% of both groups of patients were in clinical remission, and approximately half of the patients remained on steroid therapy. Autoimmune polyendocrine syndrome–positive patients had a slightly higher median SLICC Damage Index (SDI), although this was not associated with increased disease activity.

**Conclusion:**

The prevalence of autoimmune polyendocrine syndrome among SLE patients is significantly higher than in the general population (11% *vs* 0.005%), confirming the association between autoimmune thyroiditis and SLE. However, autoimmune polyendocrine syndrome–positive patients do not appear to have a more aggressive disease or develop more complications.

Rheumatology key messages

- Autoimmune polyendocrine syndrome type 3 is significantly more frequent in SLE patients than in the general population.
- Autoimmune thyroiditis is not associated with any specific clinical phenotype of SLE.
- However, routine thyroid function monitoring is essential in SLE, due to the frequent autoimmune thyroid involvement.

## Introduction

SLE is a multi-organ autoimmune disease commonly involving joints, kidneys, skin, heart, haematologic cell lines, lungs and the CNS. Approximately half a million people in Europe and a quarter of a million people in the USA have SLE [1].

It is well known that SLE patients may be at increased risk for several comorbidities and treatment-related morbidities. In fact, patients with SLE have an almost 5-fold increased risk of death compared with the general population [2]. Studies on observational cohorts have identified infections [3], hypertension [4], dyslipidaemia [5], diabetes mellitus [4], atherosclerosis [6], coronary heart disease [7], osteoporosis, avascular bone necrosis [8] and certain types of cancer (e.g. non-Hodgkin’s lymphoma) as common causes of morbidity and mortality in SLE patients [9].

To date, few studies have assessed the association between SLE and autoimmune endocrine diseases [10–12]. A few studies have found that patients with SLE have a higher prevalence of Hashimoto’s thyroiditis (HT), Graves’ disease (GD), diabetes mellitus type 1 (T1DM) and hyperparathyroidism compared with the general population. Furthermore, patients with SLE and another endocrine disease have an increased risk of developing a second endocrinopathy [10].

However, to our knowledge, there are no data about an association between SLE and autoimmune polyendocrine syndromes. Autoimmune polyendocrine syndromes are orphan diseases (ORPHA code: 282196) characterized by the non-simultaneous association of several autoimmune diseases involving at least one endocrine organ or gland, eventually leading to organ failure [11, 13]. The original classification into four types by Neufeld *et al.* in 1980 was revised by Betterle and Zanchetta in 2003 [14].

Autoimmune polyendocrine syndrome type 1 (ORPHA: 3453), caused by various mutations in the autoimmune regulator (*AIRE*) gene on chromosome 21, is characterized by the presence of candidiasis, hypoparathyroidism, and Addison’s disease. Autoimmune polyendocrine syndrome type 2 (ORPHA: 3143) is characterized by the presence of Addison’s disease along with autoimmune thyroid diseases and/or T1DM [14]. Autoimmune thyroid diseases associated with other autoimmune diseases (excluding Addison’s disease and/or hypoparathyroidism) fall under autoimmune polyendocrine syndrome type 3 (ORPHA: 227982). This syndrome is subdivided into type 3A if associated with other endocrine diseases, type 3B if associated with gastrointestinal diseases, type 3C if associated with skin, haematopoietic system, or nervous system diseases, and type 3D if associated with rheumatic diseases. Autoimmune polyendocrine syndrome type 4 (ORPHA: 227990) includes all the various clinical combinations of autoimmune diseases not included in the previous groups and affecting an endocrine organ (excluding Addison’s disease, thyroid diseases, or hypoparathyroidism) in combination with at least one more endocrine or non-endocrine organ [14].

The primary objective of this study was to describe the prevalence of autoimmune polyendocrine syndromes in a cohort of SLE patients referred to our centre. The secondary aim was to compare SLE patients with and without autoimmune polyendocrine syndromes and to assess whether the presence of autoimmune polyendocrine syndromes may predict higher disease activity or worse outcomes.

## Methods

As previously stated, the primary objective of this study was to determine the prevalence of autoimmune polyendocrine syndromes among patients with SLE. To achieve this, we retrospectively analysed the medical records of all SLE patients who were followed at our centre between 2021 and 2023.

A total of 417 adult patients (≥18 years) with a confirmed diagnosis of SLE, capable of providing informed consent, and with at least one clinical evaluation during the study period (2021–2023) were included. All the patients enrolled in the study met at least one set of SLE classification criteria from either the SLICC 2012 or the EULAR/ACR 2019 [15, 16]. Within this cohort, patients affected by autoimmune polyendocrine syndromes were identified using the ORPHA code definitions (ORPHA 282196, 3453, 3143, 227982, 227990). The screened endocrine autoimmune diseases in addition to SLE were HT, GD, T1DM, Addison’s disease, chronic mucocutaneous candidiasis, chronic hypoparathyroidism, primary ovarian insufficiency and *AIRE* gene mutation. Endocrine diseases were diagnosed according to current classification criteria [17–21]. In patients with autoimmune polyendocrine syndromes (autoimmune polyendocrine syndromes–positive patients), the presence of additional autoimmune forms without endocrine involvement was investigated.

A total of 185 patients with SLE without autoimmune polyendocrine syndromes (autoimmune polyendocrine syndrome–negative patients), randomly enrolled, served as controls. For autoimmune polyendocrine syndrome–positive and autoimmune polyendocrine syndrome–negative patients, data on the age of onset and age at diagnosis of each autoimmune disease; demographic, clinical and serological features; and current medical treatment were recorded. The analysis of current therapies included CS treatment (mg/kg at the last clinical evaluation), antimalarials (HCQ) and DMARDs, including biologic drugs such as belimumab, anifrolumab and rituximab.

Cumulative SLE damage was measured using the SLICC Damage Index (SDI) [22]; disease activity was assessed using the SLEDAI-2K [23] and SLE Disease Activity Score (SLEDAS) [24]. The 2021 Definition Of Remission In SLE (DORIS) criteria were used to identify patients in disease remission [25].

For all patients, data on ANA, anti-dsDNA, anti-ENA, LA, IgG/IgM aCL, IgG/IgM anti-beta2 glycoprotein I (aB2GPI), RF, ACPA, and direct Coombs test results were collected. ANA testing was performed using the IF technique with Hep-2 epithelial cell substrate. Anti-ENA were initially assessed by ELISA using the anti-ENA IgG ELISA kit, with confirmation via immunoblot. Anti-dsDNA was detected by chemiluminescence immunoassay, with confirmation by the IF technique using *Crithidia luciliae* as a substrate. IgG/IgM aCL and IgG/IgM aB2GPI were detected using the chemiluminescence immunoassay. LA tests were performed according to the International Society on Thrombosis and Haemostasis (ISTH) guidelines, using diluted Russell Viper Venom Time (dRVVT) and Silica Clotting Time as screening tests. The direct Coombs test was performed using monoclonal anti-human globulin.

Patients with thyroid disorders were assayed for TSH and T4, measured by chemiluminescence; normal TSH values ranged between 0.3–4.0 mIU/ml and T4 between 0.7–1.8 ng/dl. Serum anti-thyroid antibodies, anti-thyroglobulin antibodies (TgAb) and anti-thyroid peroxidase antibodies (TPOAb) were assessed.

### Statistical analysis

The database was formatted using Microsoft Excel^®^ software and subsequently imported into IBM SPSS^®^ software version 28.0.1 (IBM SPSS Inc., Chicago, Illinois). The use of Stata^®^ software version 17.0 (Stata Corporation, College Station, Texas) was also considered for test result comparisons. Normality of the distributions was assessed using the Kolmogorov–Smirnov test. Categorical variables were presented as frequencies or percentages and compared using the χ^2^ test; associations in the crosstabs were verified using standardized adjusted residuals. Continuous variables were presented as medians, IQR, and range (in the case of a skewed distribution) and compared using the Mann–Whitney test. Logistic regressions were also used to calculate the associations between predictor covariates and each of the outcome variables. A two-sided alpha level of 0.05 was considered significant for all tests. The authors had full access to and take full responsibility for the integrity of the data.

## Results

Between 2021 and 2023, a total of 417 patients were evaluated at our Rheumatology and Immunology Unit.

Forty-seven of the 417 (11%) patients (45 females and 2 males) were diagnosed as having autoimmune polyendocrine syndromes. Forty-five patients (43 women and 2 men) were affected by autoimmune polyendocrine syndrome type 3, and 2 women by autoimmune polyendocrine syndrome type 4; no patients were diagnosed with autoimmune polyendocrine syndrome type 1 or autoimmune polyendocrine syndrome type 2. After analysing for autoimmune diseases in addition to SLE in patients with autoimmune polyendocrine syndrome (autoimmune polyendocrine syndrome–positive patients) 39 cases of HT, 6 of GD, 3 of T1DM, 1 of multiple sclerosis, 1 of autoimmune urticaria (AU), 1 of autoimmune atrophic gastritis, 1 of RA, and 1 of vitiligo were found.

The autoimmune polyendocrine syndrome–positive patients at the time of data collection had a median age of 54 years (IQR 41–65), the median age of onset of SLE was 28 years (IQR 23–43) and the median disease duration was 21 years (IQR 11–24). All patients except one were of Caucasian ethnicity, and the non-Caucasian patient was of African ethnicity.

The median age in years at first autoimmune manifestation onset was 29 (IQR 21–43), 35 (IQR 26–49) at second, 27.5 (IQR 25–32.5) at third, and 35.5 at fourth. The median latency between the first and second manifestations was 3 years (IQR 1–10), 1.5 years (IQR 0.25–0.45) between the second and third, and 5 years (IQR 0.75–7.25) between the third and fourth.

Table 1 shows the sequence in which the autoimmune diseases manifested. SLE was the first manifestation of autoimmune polyendocrine syndrome in 22 patients (47%), HT was the first autoimmune manifestation for 21 (45%) patients, GD was the first for 2 (4%) patients, and 2 women started with RA (2%) and AU (2%), respectively. SLE was the second manifestation in 23 (49%) patients and the fourth for 2 (4%) patients. Forty-three patients had two concurrent autoimmune diseases; only 4 women presented with more than two autoimmune forms (their clinical and serological features are described in Table 2).

**Table 1.** Sequence of autoimmune disease presentation in patients with SLE and autoimmune polyendocrine syndromes

First disease, *n* (%)	Median age in years at first disease onset	Second disease, *n* (%)	Median age in years at second disease onset	Third disease, *n* (%)	Median age in years at third disease onset	Fourth disease, *n* (%)	Age in years at fourth disease onset
HT, 21 (45)	32	SLE, 20 (43)	35	–	–	–	–
		Vitiligo, 1 (2)	33	Atrophic gastritis, 1 (2)	34	SLE, 1 (2)	43
SLE, 22 (47)	28	HT, 15 (32)	35	–	–	–	–
		GD, 4 (9)	51.5	–	–	–	–
		T1DM, 2 (4)	30	–	–	–	–
		Multiple sclerosis, 1 (2)	26	HT, 1 (2)	28	–	–
GD, 2 (4)	42	SLE, 2 (4)	50.5	–	–	–	–
RA, 1 (2)	21	HT, 1 (2)	27	T1DM, 1 (2)	27	SLE, 1 (2)	28
AU, 1 (2)	8	SLE, 1 (2)	19	HT, 1 (2)	24	–	–

HT: Hashimoto thyroiditis; GD: Graves’ disease; RA: Rheumatoid Arthritis; T1DM: type 1 diabetes mellitus; AU: autoimmune urticaria

**Table 2.** Clinical and serological features of the four autoimmune polyendocrine syndrome–positive women presenting with more than two autoimmune forms

	First manifestation (age in years)	Second manifestation (age in years)	Third manifestation (age in years)	Fourth manifestation (age in years)	Clinical features	Serological profile	SLEDAI	SDI
**#1**	HT (20)	Vitiligo (26)	Atrophic gastritis (27)	SLE (28)	Malar rash; discoid lupus; photosensitivity; RP; sicca syndrome; asthenia; arthralgias; leukopenia; lymphopenia	ANA+ dsDNA+ Ro/SSA+ C3–C4 ↓	2	0
**#2**	RA (21)	HT (29)	T1DM (29)	SLE (30)	Livedo reticularis; fever; asthenia; erosive arthritis; pericarditis; pleuritis; glomerulonephritis; haemolytic anaemia; leukopenia; osteoporosis with fractures	ANA+ dsDNA+ Ro/SSA+ C3–C4↓ Coombs+ ACPA+ RF+ aCL IgM+	0	3
**#3**	SLE (18)	Multiple sclerosis (31)	HT (30)	–	Malar rash; asthenia; arthralgias; deforming arthritis; tenosynovitis; depression; venous thrombosis	ANA+ dsDNA+ Anti-ribosomal P protein+ C3–C4 ↓ ACPA+ RF+ LA+	0	1
**#4**	Autoimmune urticaria (8)	SLE (19)	HT (24)	–	Malar rash; photosensitivity; asthenia; glomerulonephritis	ANA+ dsDNA+ C3–C4 ↓ aCL IgG+ aB2GPI IgG+	0	0

HT: Hashimoto thyroiditis; T1DM: type 1 diabetes mellitus; aB2GPI:anti-B2glycoprotein I.

The data for 185 autoimmune polyendocrine syndrome–free patients [179 (97%) females and 6 (3%) males] were compared with those with autoimmune polyendocrine syndromes. Autoimmune polyendocrine syndrome–free patients (autoimmune polyendocrine syndrome–negative patients) at the time of recruitment had a median age of 52 years (IQR 42–62), a median disease duration of 23 years (IQR 15–32) and a median age at onset of SLE of 27 years (IQR 20–34). One hundred and seventy-six (95%) patients were of Caucasian ethnicity, 2 (1%) of African ethnicity and 7 (4%) of Asian ethnicity. Table 3 compares the epidemiological data, tobacco exposure and BMIs of the two populations.

**Table 3.** Epidemiological features of SLE patients with and without autoimmune polyendocrine syndromes

	SLE with autoimmune polyendocrine syndromes	SLE without autoimmune polyendocrine syndromes	*P* value
Total SLE Patients	47	185	–
Age in years, median (IQR)	54 (41–65)	52 (42–62)	0.930
Age in years at SLE onset, median (IQR)	28 (23–43)	27 (20–34)	0.047
Female, *n* (%)	45 (96)	179 (97)	0.734
Disease duration in years, median (IQR)	21 (11–24)	23 (15–32)	0.019
Tobacco exposure *n* (%)	15 (32)	53 (29)	0.660
BMI, median (IQR)	24 (22–28)	22 (21–26)	0.251

A comparison of the various cumulative clinical disease domains between SLE patients with and without autoimmune polyendocrine syndromes was made. Forty (85%) autoimmune polyendocrine syndrome–positive and 148 (80%) autoimmune polyendocrine syndrome–negative patients presented constitutional symptoms (*P = *0.546); 37 (79%) autoimmune polyendocrine syndrome–positive and 158 (85%) autoimmune polyendocrine syndrome–negative patients presented cutaneous manifestations (*P = *0.264); 40 (85%) autoimmune polyendocrine syndrome–positive and 171 (92%) autoimmune polyendocrine syndrome–negative patients developed musculoskeletal symptoms (*P = *0.118); 15 (32%) autoimmune polyendocrine syndrome–positive and 52 (28%) autoimmune polyendocrine syndrome–negative patients had neurological involvement (*P = *0.607); 11 (23%) autoimmune polyendocrine syndrome–positive and 33 (18%) autoimmune polyendocrine syndrome–negative patients developed at least one form of serositis (*P = *0.385); 26 (55%) autoimmune polyendocrine syndrome–positive and 119 (64%) autoimmune polyendocrine syndrome–negative patients had haematological involvement (*P = *0.255); 12 (26%) autoimmune polyendocrine syndrome–positive and 71 (38%) autoimmune polyendocrine syndrome–negative patients had renal involvement (*P = *0.120); 15 (32%) autoimmune polyendocrine syndrome–positive and 44 (24%) autoimmune polyendocrine syndrome–negative patients developed cardiac manifestations (*P = *0.253); 6 (13%) autoimmune polyendocrine syndrome–positive and 27 (15%) autoimmune polyendocrine syndrome–negative patients had pulmonary involvement (*P = *0.749); no autoimmune polyendocrine syndrome–positive patients and 6 (3%) autoimmune polyendocrine syndrome–negative patients had gastrointestinal manifestations; 12 (25%) autoimmune polyendocrine syndrome–positive and 31 (17%) autoimmune polyendocrine syndrome–negative patients developed ocular pathology (*P = *0.167); 5 (11%) autoimmune polyendocrine syndrome–positive and 15 (8%) autoimmune polyendocrine syndrome–negative patients had at least one thrombotic event (*P = *0.596). Secondary APS was diagnosed in 6 (13%) autoimmune polyendocrine syndrome–positive and 23 (12%) autoimmune polyendocrine syndrome–negative patients, *P = *0.911.

A significant statistical association was found between polyglandular syndromes and both pulmonary hypertension (*P = *0.044) and renal microangiopathy (*P = *0.044). In contrast, autoimmune polyendocrine syndrome–positive patients appeared to have less joint involvement and a lower incidence of arthritis (*P = *0.009). The details of the various clinical manifestations, categorized by SLE domain, are presented in Table 4.

**Table 4.** Clinical manifestation in SLE patients with and without autoimmune polyendocrine syndromes

	SLE with autoimmune polyendocrine syndromes	SLE without autoimmune polyendocrine syndromes	*P*-value		SLE with autoimmune polyendocrine syndromes	SLE without autoimmune polyendocrine syndromes	*P*-value
Total SLE patients, *n* (%)	47	185	–	Total SLE patients, *n* (%)	47	185	–
**Constitutional symptoms, *n* (%)**	40 (85)	148 (80)	0.546	**Haematological involvement, *n* (%)**	26 (55)	119 (64)	0.255
Fever, *n* (%)	15 (32)	75 (41)	0.267	Haemolytic anaemia, *n* (%)	10 (21)	30 (16)	0.412
Asthenia, *n* (%)	39 (83)	137 (75)	0.221	Leukopenia, *n* (%)	18 (38)	96 (52)	0.096
				Thrombocytopenia, *n* (%)	12 (25)	28 (15)	0.092
**Cutaneous manifestations, *n* (%)**	37 (79)	158 (85)	0.264	Lymphocytopenia, *n* (%)	11 (23)	63 (34)	0.162
Butterfly rash, *n* (%)	24 (51)	108 (58)	0.366	Neutropenia, *n* (%)	6 (13)	20 (11)	0.704
scLE, *n* (%)	1 (2)	15 (8)	0.148				
Discoid rash, *n* (%)	16 (34)	76 (41)	0.378	**Renal involvement, *n* (%)**	12 (26)	71 (38)	0.120
Tumid lupus, *n* (%)	1 (2)	1 (0,5)	0.293	Renal failure, *n* (%)	2 (4)	4 (2)	0.419
Alopecia, *n* (%)	12 (25)	71 (38)	0.101	Glomerulonephritis Class I, *n* (%)	0	1 (0,5)	0.613
Photosensitivity, *n* (%)	20 (43)	96 (52)	0.253	Glomerulonephritis Class II, *n* (%)	2 (4)	8 (4)	0.983
Oral ulcers, *n* (%)	0	4 (2)	0.313	Glomerulonephritis Class III, *n* (%)	2 (4)	14 (8)	0.424
Cutaneous vasculitis, *n* (%)	4 (9)	37 (20)	0.070	Glomerulonephritis Class IV, *n* (%)	4 (8)	27 (15)	0.274
Sicca symptoms, *n* (%)	15 (32)	49 (26)	0.457	Glomerulonephritis Class V, *n* (%)	3 (6)	14 (8)	0.781
RP, *n* (%)	20 (43)	76 (41)	0.855	Glomerulonephritis Class VI, *n* (%)	1 (2)	0	0.047
				Tubulointerstitial nephritis, *n* (%)	0	1 (0,5)	0.613
**Musculoskeletal involvement, *n* (%)**	40 (85)	171 (92)	0.118	Microangiopathy, *n* (%)	2 (4)	1 (0,5)	0.044^b^
Arthralgias, *n* (%)	37 (79)	159 (86)	0.222				
Arthritis, *n* (%)	20 (49)	128 (69)	0.009^a^	**Cardiac involvement, *n* (%)** (including pericarditis)	15 (32)	44 (24)	0.253
Jaccoud arthropathy, *n* (%)	3 (6)	14 (8)	0.781	Myocarditis, *n* (%)	1 (2)	2 (1)	0.571
Osteonecrosis, *n* (%)	0	2 (1)	0.474	Endocarditis, *n* (%)	5 (11)	16 (9)	0.671
Myositis, *n* (%)	0	5 (3)	0.255	Myocardial infarction, *n* (%)	2 (4)	7 (4)	0.881
				Cardiac arrhythmias, *n* (%)	0	6 (3)	0.211
**Neurological involvement, *n* (%)**	15 (32)	52 (28)	0.607				
Headache, *n* (%)	3 (6)	32 (17)	0.062	**Pulmonary involvement, *n* (%)**	6 (13)	27 (15)	0.749
Seizures, *n* (%)	4 (8)	11 (6)	0.536	Pulmonary hypertension, *n* (%)	2 (4)	1 (0,5)	0.044^c^
Psychosis, *n* (%)	1 (2)	2 (1)	0.571	Lupus pneumonia, *n* (%)	0	2 (1)	0.474
Depression, *n* (%)	7 (15)	15 (8)	0.156	Interstitial lung disease, *n* (%)	1 (2)	3 (2)	0.812
Confusion, *n* (%)	1 (2)	1 (0,5)	0.293	Shrinking lung, *n* (%)	0	2 (1)	0.474
Cerebral ischaemia/stroke, *n* (%)	1 (2)	5 (3)	0.824				
Transient ischaemic stroke, *n* (%)	2 (4)	14 (8)	0.424	**Gastroenterological involvement, *n* (%)**	0	6 (3)	0.211
Cognitive impairment, *n* (%)	1 (2)	10 (5)	0.345	Pancreatitis, *n* (%)	0	1 (0,5)	0.613
Peripheral mononeuropathy, *n* (%)	0	4 (2)	0.309	Oesophageal dyskinesia, *n* (%)	0	3 (2)	0.380
Peripheral polyneuropathy, *n* (%)	1 (2)	7 (4)	0.578	Mesenteric vasculitis, *n* (%)	0	2 (1)	0.454
Cranial nerve neuropathy, *n* (5%)	1 (2)	2 (1)	0.571				
				**Ocular involvement, *n* (%)**	12 (25)	31 (17)	0.167
**Serositis, *n* (%)**	11 (23)	33 (18)	0.385				
Pleuritis, *n* (%)	5 (11)	19 (10)	0.941	**Thrombosis, *n* (%)**	5 (11)	15 (8)	0.596
Pericarditis, *n* (%)	10 (21)	25 (13)	0.184				

a OR 0.43, 95% CI 0.22–0.82;

b OR 8.18, 95% CI 0.72–92.19;

c OR 8.18, 95% CI 0.72–92.19.

All patients tested positive for ANA at least once. Forty-two (89%) autoimmune polyendocrine syndrome–positive and 177 (96%) autoimmune polyendocrine syndrome–negative patients had positive anti-dsDNA antibodies (*P = *0. 093); anti-ENA antibodies were positive in 29 (62%) autoimmune polyendocrine syndrome–positive and 113 (62%) autoimmune polyendocrine syndrome–negative patients (*P = *0.938); aPL were positive for 20 (43%) autoimmune polyendocrine syndrome–positive and 92 (50%) autoimmune polyendocrine syndrome–negative patients (*P = *0.362); the LA test was positive for 9 (19%) autoimmune polyendocrine syndrome–positive and 45 (25%) autoimmune polyendocrine syndrome–negative patients (*P = *0.432). The serological details for the two groups are provided in Table 5.

**Table 5.** Serological data for SLE patients with and without autoimmune polyendocrine syndromes

	SLE with APS	SLE without APS	*P*-value
Total SLE patients, *n* (%)	47	185	–
ANA, *n* (%)	47 (100)	185 (100)	–
Anti-dsDNA, *n* (%)	42 (89)	177 (96)	0.093
Anti-ENA, *n* (%)	29 (62)	113 (61)	0.938
Anti-Ro/SSA, *n* (%)	18 (38)	80 (43)	0.540
Anti-La/SSB, *n* (%)	4 (9)	15 (8)	0.928
Anti-Sm, *n* (%)	8 (17)	38 (20)	0.589
Anti-ribosomal P protein, *n* (%)	3 (6)	4 (2)	0.131
Anti-U1RNP, *n* (%)	5 (11)	46 (25)	0.035^a^
Anti-Ki, *n* (%)	3 (6)	3 (2)	0.066
Anti-nucleosome, *n* (%)	3 (6)	3 (2)	0.066
Anti-histone, *n* (%)	2 (4)	3 (2)	0.267
Anti-Scl70, *n* (%)	1 (2)	2 (1)	0.571
Anti-CENP, *n* (%)	0	2 (1)	0.474
Anti-Jo1, *n* (%)	0	2 (1)	0.474
AMA, *n* (%)	1 (2)	2 (1)	0.571
aPL positivity, *n* (%)	20 (43)	92 (50)	0.362
aCL IgG, *n* (%)	11 (23)	49 (27)	0.653
aCL IgM, *n* (%)	5 (11)	31 (17)	0.295
aB2GPI IgG, *n* (%)	8 (17)	56 (30)	0.067
aB2GPI IgM, *n* (%)	4 (9)	31 (17)	0.155
LA test, *n* (%)	9 (19)	45 (25)	0.432
aPL single positivity, *n* (%)	11 (23)	34 (18)	0.436
aPL double positivity, *n* (%)	6 (13)	34 (18)	0.363
aPL triple positivity, *n* (%)	3 (6)	25 (13)	0.180
RF, *n* (%)	3 (6)	22 (16)	0.459
ACPA, *n* (%)	3 (6)	7 (9)	0.193
Coombs test, *n* (%)	13 (28)	72 (51)	0.242
C3 consumption, *n* (%)	31 (66)	159 (86)	0.001^b^
C4 consumption, *n* (%)	35 (75)	159 (86)	0.058

a OR 0.36 95% CI 0.13–0.96;

b OR 0.32 95% CI 0.15–0.66. aB2GPI: anti-B2glycoprotein I.

At the last clinical evaluation, autoimmune polyendocrine syndrome–positive patients had a median SLEDAI of 0 (range 0–8) and a median SLEDAS of 0.37 (range 0–22.7). Thirty-eight (81%) autoimmune polyendocrine syndrome–positive patients were in clinical remission. Twenty-three (49%) patients were still receiving CS treatment (median prednisone equivalent of 25 mg per week). Thirty-eight (81%) patients were on HCQ, and 21 (45%) were receiving DMARD therapy. The median SDI was 1 (range 0–5). In the autoimmune polyendocrine syndrome–negative control group, the median SLEDAI was 2 (range 0–10) and the median SLEDAS was 1.12 (range 0–20.1). One hundred and fifty-two (83%) patients were in clinical remission. Eighty-five (46%) were using CSs (median prednisone equivalent of 25 mg per week), 146 (80%) were on HCQ, and 102 (56%) were receiving DMARDs. A comparison of disease activity, clinical remission, ongoing therapy, and cumulative damage at the last evaluation is shown in Table 6. Notably, the last clinometric indices SLEDAI (*P = *0.002) and SLEDAS (*P = *0.038) were significantly lower in autoimmune polyendocrine syndrome–positive patients.

**Table 6.** Damage, disease activity, and current therapy at the last evaluation in SLE patients with and without autoimmune polyendocrine syndromes

	SLE with autoimmune polyendocrine syndromes	SLE without autoimmune polyendocrine syndromes	*P*-value
Total SLE patients, *n* (%)	47	185	–
SLEDAI, median (min–max)	0 (0–8)	2 (0–10)	0.002
SLEDAS, median (min–max)	0,37 (0–22,7)	1,12 (0–20,1)	0.038
SDI, median (min–max)	1 (0–5)	0 (0–6)	0.669
DORIS remission, *n* (%)	38 (81)	152 (83)	0.665
CSs, *n* (%)	23 (49)	85 (46)	0.714
Prednisone mg/wk, median (min–max)	0 (0–75)	0 (0–70)	0.538
HCQ, *n* (%)	38 (81)	146 (80)	0.870
DMARDs, *n* (%)	21 (45)	102 (56)	0.175

DORIS: Definition Of Remission In SLE; SDI: SLICC Damage Index; SLEDAS: Systemic Lupus Erythematosus Disease Activity State.

## Discussion

In the last 20 years, the number of individuals affected by autoimmune diseases has increased, especially in more economically developed countries [26]. Several mechanisms involving genetic and environmental risk factors have been proposed to explain the increased prevalence of the autoimmune forms. Autoimmune diseases often share common predisposing factors, including genetic, environmental and behavioural determinants. Most of the autoimmune diseases observed show a prevalence in females [27, 28], major genetic risk factors, partially overlapping cytokine profiles, and lifestyle-related risk factors. The most prominent genetic risk factors are alleles within the HLA class, particularly HLA-DRB1 [29], while a large number of non-HLA genes (*FLT3*, *IRF5*, *STA4*) involved in autoimmune diseases are interlinked in a network that regulates IFN signalling and dendritic cell and T cell function [30, 31]. PTPN22 also codes for a protein involved in both T and B cell signalling, with a strong association with autoimmune forms [32].

Although genetics play an important role, environmental factors have been investigated as contributors to autoimmune disease development, particularly smoking, occupational exposure, alcohol consumption, diet, obesity, stress with high levels of cortisol, low vitamin D levels, and viral infections [33]. Additionally, it is known that the presence of autoantibodies and their related autoimmune disease predispose patients to manifest non-specific antibodies for the disease, in a process called polyautoimmunity [34].

A close relationship between the immune and endocrine systems is well established [35, 36]. Few observational studies and meta-analyses have shown a high prevalence of autoimmune endocrine diseases, in particular thyroiditis and hyperparathyroidism, in SLE patients [11]. However, to the best of our knowledge, no case–control studies have analysed the prevalence and characteristics of SLE patients with autoimmune polyendocrine syndromes.

In our hospital, 11% of SLE patients currently under follow-up have another autoimmune disease affecting the glands that allows the diagnosis of autoimmune polyendocrine syndrome. Specifically, 9% of SLE patients were diagnosed with HT, 1.5% with GD, and 0.5% with T1DM.

Autoimmune polyendocrine syndromes are characterized by autoantibodies against antigens of certain endocrine organs and by the presence of lymphocytic infiltrates. Autoimmunity directed towards these organs results in antibody-dependent cytotoxicity, similar to what occurs in SLE target tissues [11].

Autoimmune polyendocrine syndrome type 1 is a rare monogenic disease caused by loss-of-function mutations in the *AIRE* gene, which result in the loss of thymic tolerance of T lymphocytes. The clinical characteristics of autoimmune polyendocrine syndrome type 1 include the classic triad of chronic mucocutaneous candidiasis, primitive hypoparathyroidism, and adrenal insufficiency. To these manifestations can be added any other autoimmune form not of endocrinological relevance, including rheumatological diseases. Autoimmune polyendocrine syndrome type 1 usually manifests in infancy or early childhood, with a prevalence of 1/100 000/year [37]. However, in our cohort, there were no cases of type 1 autoimmune polyendocrine syndrome, and there is no literature about the prevalence of type 1 autoimmune polyendocrine syndrome in cohorts of SLE patients.

Autoimmune polyendocrine syndrome type 2 is characterized by the presence of adrenal insufficiency associated with T1DM and/or an autoimmune thyroid disorder. To this can be added any other endocrine or non-endocrine pathology of autoimmune origin. Autoimmune polyendocrine syndrome type 2 is five times more common in women and has a prevalence of 10–20/100 000 [37]. In autoimmune polyendocrine syndrome type 2, Addison’s disease is generally the first disorder to manifest, often misrecognized. Only a few case reports of patients with SLE and autoimmune polyendocrine syndrome type 2 exist in the literature [38, 39]. It is known that specific haplotypes (HLA DR3 and DR4) are more common in SLE and autoimmune polyendocrine syndrome type 2 and that, although not directly involved in the pathogenesis of the disease, they result in the production of certain pathogenic autoantibodies, but a clear association between autoimmune polyendocrine syndrome type 2 and systemic autoimmune disease has never been established. In our cohort of SLE patients, there were no cases of Addison’s disease, and therefore no patients were diagnosed with autoimmune polyendocrine syndrome type 2. This might suggest that SLE is not associated with an increased risk of adrenal insufficiency of autoimmune origin compared with the general population.

Autoimmune polyendocrine syndrome type 3 is characterized by the presence of autoimmune thyroid disease associated with another autoimmune form [40]. In the study by McDonagh *et al.*, up to 30% of SLE patients had at least one comorbid autoimmune disorder, of which 4% had HT and 4% had GD, and in most cases, comorbid autoimmune forms occur after SLE diagnosis [41]. Posselt *et al.* observed an HT frequency in SLE patients of 12%, twice as high as in the control group. Moreover, in this study, SLE and HT patients had a butterfly rash less often and anti-Sm positivity more often than controls [42]. Pyne *et al.* found an incidence rate of thyroid antibodies ranging from 14% to 68% in the population with isolated SLE [43]. There are also researchers who have reported that the levels of TgAb correlated with anti-dsDNA antibody levels in SLE patients [44]. In our cohort, 45/47 (96%) patients were diagnosed with autoimmune polyendocrine syndrome type 3: 39/45 (87%) patients had HT and 6/45 (13%) had GD. Autoimmune polyendocrine syndrome type 3 was the most common form in our population. This is consistent with the literature, which reports a high prevalence of autoimmune thyroiditis in SLE patients [45]. For 24/45 (53%) patients, the onset disease was thyroid pathology, while for 21 (47%) patients, SLE manifested before HT or GD.

Autoimmune polyendocrine syndrome type 4 includes all the various clinical combinations of autoimmune diseases not included in the previous groups [40]. In our population, only 2/47 (4%) patients were diagnosed with autoimmune polyendocrine syndrome type 4, and both were affected by T1DM. For these patients, the diagnosis of SLE slightly preceded the diagnosis of T1DM. These data seem to support the literature suggesting that there is no clear association between autoimmune diabetes and SLE [10, 46].

Comparison of the clinical and laboratory data between autoimmune polyendocrine syndrome–positive and autoimmune polyendocrine syndrome–negative patients revealed no significant differences. autoimmune polyendocrine syndrome–positive patients did not show a higher incidence of severe SLE-related manifestations, such as renal disease, neurological involvement, or cardiac issues. The only conditions statistically associated with autoimmune polyendocrine syndromes were renal microangiopathy and pulmonary hypertension. However, given the low number of affected patients and the rarity of these conditions, no definitive conclusions can be drawn.

At the last evaluation, ∼80% of both groups of patients were in clinical remission. Approximately half of the patients were still on steroid therapy at the time of the last evaluation, but with a daily prednisone-equivalent dosage of <5 mg/day. Additionally, the autoimmune polyendocrine syndrome–positive patients had a slightly higher median SDI than autoimmune polyendocrine syndrome–negative patients, although this was not associated with increased disease activity.

In conclusion, the incidence of autoimmune polyendocrine syndrome type 3 among SLE patients in our cohort was significantly higher than in the general Caucasian population (11% *vs* 0.005%) [47], highlighting the fact that thyroid autoimmune disorders are quite prevalent in patients with SLE. Our findings support the existing literature [12] regarding the association between autoimmune thyroiditis and SLE, presumably correlated with the common Th1 immune predominance as a pathogenetic basis in the two diseases [48, 49].

Our study did not indicate substantial differences between SLE patients with and without autoimmune polyendocrine syndrome. Autoimmune polyendocrine syndrome–positive patients did not appear to experience a more aggressive disease course or a higher incidence of disease-related complications.

However, the high frequency of autoimmune thyroiditis underscores the clinical importance of monitoring thyroid function in SLE patients. Any signs, symptoms, or laboratory abnormalities suggestive of thyroid dysfunction should be carefully evaluated, as undiagnosed thyroid disease could significantly impact patient well-being. While no notable differences in lupus disease manifestations were observed between autoimmune polyendocrine syndrome–positive and autoimmune polyendocrine syndrome–negative patients, enhanced thyroid function monitoring remains crucial in this high-risk population.

Routine screening for other autoimmune endocrine diseases does not appear necessary, as our cohort did not reveal an association between SLE and autoimmune polyendocrine syndrome types 1, 2 or 4. However, given the rarity of these syndromes, definitive conclusions cannot be established. The limitations of this study primarily arise from the small number of autoimmune polyendocrine syndrome–positive patients, which restricts the robustness of the statistical analysis. Additionally, disease activity and CS use were assessed solely based on data from the last evaluation, lacking the benefit of longitudinal patient follow-up data. To gain a more comprehensive understanding of the relationship between autoimmune polyendocrine syndromes and SLE, further studies and multicentric research are necessary.

The study involving human participants (ASST_BS_CLIN_PZ_SPA-BS) was reviewed and approved by Ethics Committee of Brescia (approval number No. 5517). Each patient received and signed a dedicated informed consent form.

## Data Availability

The raw data supporting the conclusions of this article will be made available by the authors, without undue reservation.
